# Genome-Wide Survey Indicates Diverse Physiological Roles of *Dendrobium officinale* Calcium-Dependent Protein Kinase Genes

**DOI:** 10.3390/ijms23031298

**Published:** 2022-01-24

**Authors:** Xingyu Yang, Zhiyu Chen, Xin Yin, Yuhua Wang, Yunqiang Yang, Yongping Yang

**Affiliations:** 1 The Germplasm Bank of Wild Species, Kunming Institute of Botany, Chinese Academy of Sciences, Kunming 650201, China; yangxingyu@mail.kib.ac.cn (X.Y.); chenzhiyu@mail.kib.ac.cn (Z.C.); yinxin@mail.kib.ac.cn (X.Y.); 2 Institute of Tibetan Plateau Research at Kunming, Kunming Institute of Botany, Chinese Academy of Sciences, Kunming 650201, China; 3 Key Laboratory of Economic Plants and Biotechnology, Kunming Institute of Botany, Chinese Academy of Sciences, Kunming 650201, China; wangyuhua@mail.kib.ac.cn; 4 Academy of Biological Sciences, University of Chinese Academy of Sciences, Beijing 100049, China

**Keywords:** *Dendrobium officinale*, *CDPK* gene family, expression pattern, functional divergence

## Abstract

Calcium-dependent protein kinases (CDPKs) are crucial calcium ions (Ca^2+^) sensors in plants with important roles in signal transduction, plant growth, development, and stress responses. Here, we identified 24 genes encoding *CDPK*s in *Dendrobium officinale* using genome-wide analysis. The phylogenetic analysis revealed that these genes formed four groups, with similar structures in the same group. The gene expression patterns following hormone treatments and yeast two-hybrid of homologous *CDPK* gene pairs with *Rbohs* showed differences, indicating functional divergence between homologous genes. In addition, the rapid accumulation of hydrogen peroxide (H_2_O_2_) and stomatal closure was observed in response to salicylic acid (SA)/jasmonic acid (JA) stress. Our data showed that CDPK9-2 and CDPK20-4 interacted with Rboh D and Rboh H, respectively, and were implicated in the generation of H_2_O_2_ and regulation of the stomatal aperture in response to salicylic acid/jasmonic acid treatment. We believe these results can provide a foundation for the functional divergence of homologous genes in *D. officinale*.

## 1. Introduction

Because plants are immobile, they have evolved several mechanisms and signaling networks to recognize and resist various stresses, such as drought, salinity, and pathogen attack [1]. As one of the most widely studied second messengers, calcium ions (Ca^2+^) play an indispensable role in signaling pathways [2]. Signal transduction pathways mediated by Ca^2+^ are known to target different proteins functioning in various biological processes. For example, signal transduction pathways targeting enzymes can affect cell metabolism, whereas those targeting cytoskeletal proteins affect cell morphology and movement. Three types of Ca^2+^-binding proteins, namely calmodulins (CaMs), calmodulin-like proteins, and calcineurin B-like proteins, or Ca^2+^ sensors encoding calcium-dependent protein kinases (*CDPKs*) detect changes in cellular Ca^2+^ concentrations. These molecules initiate multiple reactions in response to diverse stimuli in plants [3,4,5,6]. *CDPKs* have been widely studied because they can directly bind Ca^2+^ in a CaM-independent manner. Previous studies have demonstrated that *CDPKs* are absent in humans, fungi, and insects but are widely found in plants [7,8]. CDPKs contain four important conserved domains including the N-terminal variable domain that contains palmitoylation or myristoylation sites and allows the CDPK to locate correctly. The Ser/Thr kinase domain that binds to ATP is involved in autophosphorylation; the autoinhibitory domain is speculated to be a part of the CaM-like domain; and a CaM-like regulatory domain with from one to four EF-hands has been implicated in Ca^2+^ binding [9,10,11,12,13,14,15,16,17].

*CDPKs* are abundantly present in several plant species; 28, 17, 31, and 25 *CDPK* genes are present in barley, grapevine, pepper, and maize, respectively [18,19,20,21]. Previous studies have demonstrated the responses of *CDPKs* to multiple stress stimuli. In cotton, *GrCDPK14* is significantly upregulated following 12 h of cold stress [22]. Treatment with varying concentrations of methyl jasmonate (MeJA) upregulates the expression of *RcCDPK3/7/10/11* [23]. The functional diversification of *CDPKs*, detected by other studies, depends on their interactions with different molecular targets [16,24,25]. Among them, *RcCDPK 1/4/5/13/14* interacts with *rolB/C* to regulate the formation of callus in the cultures of *Rubia cordifolia*. Tobacco *CDPK1* interacts with the repression of shoot growth (*RSG*) to regulate the gibberellin (GA) pathways [23,26]. In strawberry, FaCDPKs interact with respiratory burst oxidase homolog (Rboh), another target of CDPK to generate hydrogen peroxide (H_2_O_2_) in response to drought stress [27]. In rice, OsCDPK5/11 interacts with Rboh H, resulting in the accumulation of reactive oxygen species (ROS) under oxygen-deficient conditions [28]. Similarly, StCDPK4/5 phosphorylates StRboh to activate ROS production in response to stress in potato leaves [29].

*Dendrobium officinale* (Kimura and Migo) is a rare and precious Chinese medicinal herb that usually grows on cliffs in diverse habitats [30,31,32]. *D. officinale* is the third largest genus in the Orchidaceae family [33]. It has been exploited for its medicinal value, such as immunity-enhancing property, since the Tang dynasty [34]. Over the past 20 years, researchers have been trying to transform it from an endangered plant to one for use on an industrial scale [35]. However, these plants are still vulnerable to several stresses. Although *CDPKs* are known to regulate stress responses and resistance, their roles have not been explored in *D. officinale*. In this study, we identified *CDPK* genes in the *D. officinale* genome and analyzed their phylogenetic relationships and expression patterns. In addition, we studied their functional divergence by analyzing interactions of DoCDPKs and DoRbohs following hormone treatments. Our results revealed the functional divergence of homologous genes and the biological function of the *D. officinale CDPK* gene family.

## 2. Results

### 2.1. Identification of 24 CDPK Genes in D. officinale

Thirty-five proteins were filtered and extracted by TBtools [36]. After removing shorter alternative splice variants, 24 proteins were identified as DoCDPKs and were numbered according to the phylogenetic relationship (Figure S1) and protein identity (Table S1) with CDPKs from *Arabidopsis thaliana*. The molecular weight of CDPKs (Table 1) ranged from 35.07 kDa (DoCDPK13-2) to 65.73 kDa (DoCDP20-3). The isoelectric point was approximately 5–6.7, except for DoCDPK16, which was 8.08. The majority of DoCDPKs (18 out of 24) had four EF-hand motifs, all of which had a palmitoylation site. However, less than half of them had a myristoylation site, and only DoCDPK3-2 had an N-terminal acylation site. Homologous gene pairs displayed similar molecular weights, isoelectric points, and domain structures. For example, the molecular weights of DoCDPK20-1, 20-2, 20-3, and 20-4 were 64.82, 65.73, 63.92, and 63.92 kDa, respectively. The isoelectric points of DoCDPK20-1, 20-2, 20-3 was 5.15, except for DoCDPK20-4, which was 5.51. All four proteins had palmitoylation sites. These results suggested a similar biological role of homologous gene pairs.

### 2.2. Phylogenetic and Gene Structure Analyses of DoCDPKs

**Figure 1:**
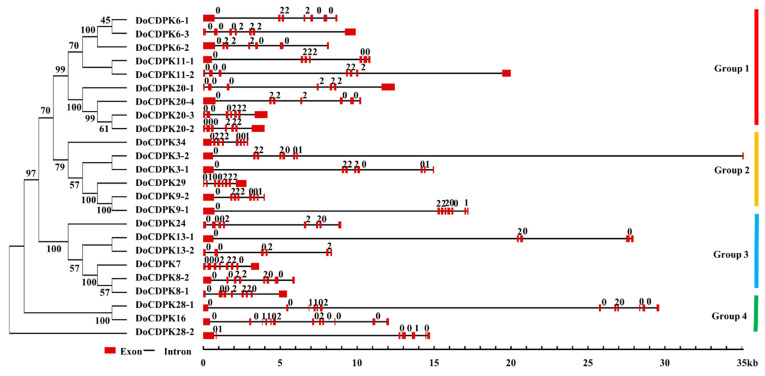
Phylogenetic relationship and gene structure of *D. officinale* CDPKs. A neighbor-joining tree was created using full-length protein sequences of 24 DoCDPKs using MEGAX 7.0 with 1000 bootstrap replicates. Numbers beside branches indicate bootstrap support. Red rectangles and black lines represent exons and introns, respectively. The number on lines indicates the intron phase. Four groups (1–4) are displayed in different colors.

Phylogenetic analysis divided the 24 *CDPK* of *D. officinale* genes into four groups (Figure 1). Group 4 had the fewest *DoCDPK* members (three), whereas the number of *CDPKs* in the other three groups ranged from six to nine. Gene structural analyses revealed that genes within the same group had similar numbers and lengths of exons. The length of introns varied within and among groups, ranging from <1 to >25 kb. In terms of intron phases, the first intron was highly conserved in phase-0; however, the majority of fourth introns were in phase-2 in genes in group 1–3. For genes in group 4, the first intron was in phase-0, and the last intron was in phase-1. The complex structure of group 4 genes could be indicative of different divergence times, suggesting functional differences between *DoCDPKs* in group 4 and those in the other three groups.

### 2.3. Natural Selection Estimation and Conserved Motif Analysis

**Figure 2:**
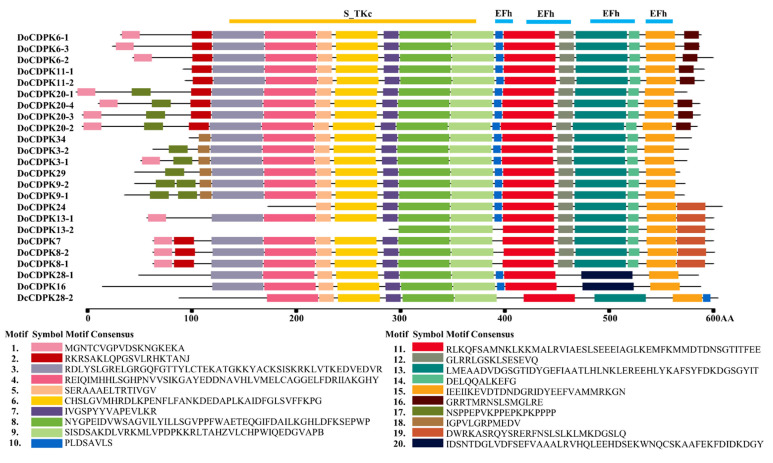
Motif organization in members of the *CDPK* gene family in *D. officinale.* Conserved motifs were predicted using the MEME software (meme-suite.org). Different motifs are marked by differently colored rectangles. Yellow and blue bar indicate conserved protein kinase domain and EF-hand motif, respectively.

To further explore the evolutionary relationships of *DoCDPKs*, *DoCDPK* homology gene pairs were compared to evaluate their phylogenetic relationships (Table 2). At the amino acid level, the protein identity of homologous gene pairs ranged from 69.60% between DoCDPK3-2 and DoCDPK3-1 to 87.53% between DoCDPK11-1 and DoCDPK11-2, except DoCDPK 13-1/2, because DoCDPK 13-2 was considerably shorter. The average identity between proteins was the highest in group 1 (82.05%) and the lowest in group 4 (70.23%). We calculated the ratio of nonsynonymous (Ka) and synonymous (Ks) substitutions. Both the Ka/Ks ratio of each pair and the average Ka/Ks ratio (0.1042) were less than 1, suggesting that DoCDPKs underwent purifying selection. Next, to gain insight into gene function, 20 conserved motifs were predicted using the MEME software (Figure 2). More than half of the detected motifs were associated with kinase and EF-hand activity. According to the predicted conserved domain analysis, the Ser/Thr protein kinase domain was composed of motifs 3–9, and the four EF-hand motifs were composed of motifs 10, 11, 13, and 15. The motifs in group 4 were conserved in every group. Motifs 12 and 14 were present in all members of group 3, and each group had at least one specific motif. The DoCDPKs in group 1 contained the highest number of motifs (up to 17), whereas those in the other three groups had approximately 15, 15, and 11 motifs, respectively. These results indicate that apart from the main functions of DoCDPKs, a certain level of functional divergence also exists.

### 2.4. Transcription Profiles of DoCDPKs in Different Tissues

**Figure 3:**
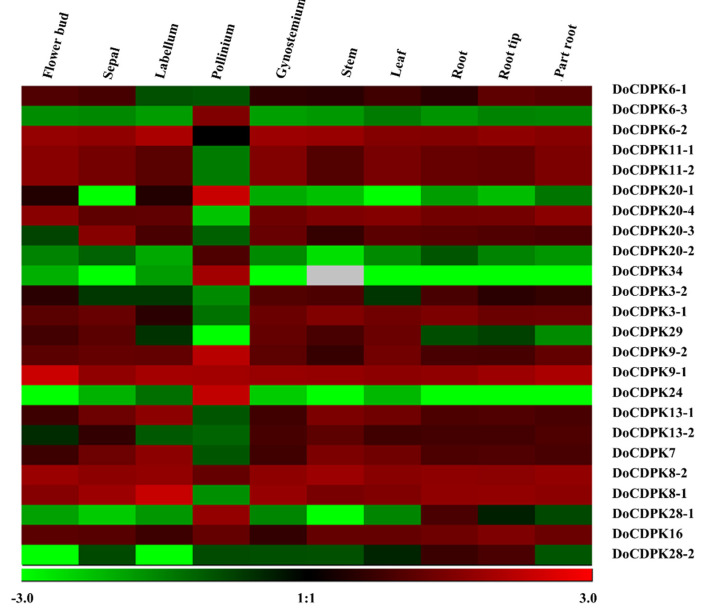
Expression patterns of *DoCDPK* genes in different organs of *D. officinale*. Data for the transcript levels of *DoCDPK*s using the FPKMs value (fragments per kilobase of exon model per million mapped fragments) downloaded from the Dendrobium gene expression database (ncku.edu.tw). A heatmap was generated using the Genesis software. Parameters were set as log 10 transformation, and genes were normalized. High, moderate, and low expression of one gene is shown in red, black, and green, respectively, in different tissues. Gray represents the FPKM value of 0.

To better understand the connection between evolution and the functional divergence of *DoCDPKs*, we analyzed the levels of gene transcript levels in different tissues (Figure 3). Certain *CDPKs* were expressed specifically in one tissue, among which *DoCDPK24* was only detected in the pollinium. Certain *DoCDPKs*, such as *DoCDPK8-2*, were detected in all 10 analyzed tissues. *DoCDPK20-1*, *29*, *13-2*, *28-1*, and *28-2* exhibited the opposite expression patterns in vegetative organs and reproductive organs, except for the pollinium, whereas others displayed the same expression pattern in vegetative and reproductive organs. Although *DoCDPK34* was not expressed in the stem, it was abundantly expressed in other tissues. Genes in the same group or closely related gene pairs often had different expression patterns. For example, the homologous genes *DoCDPK6-1*/*-2*/*-3* in group 1 showed highly different expression patterns among the tested tissues. As mentioned above, the expression of different *DoCDPK*s in different tissues reflected their diverse functions during both vegetative and reproductive growth. This result is indicative of functional divergence between homologous gene pairs.

### 2.5. Prediction of Cis-Acting Elements and Gene Expression Patterns under Different Hormone Treatments

**Figure 4:**
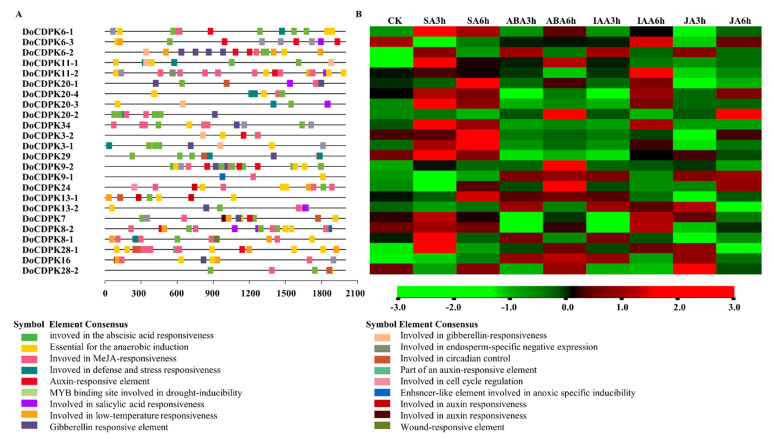
Distribution of cis-acting elements related to stress responsiveness, plant growth, and development. (**A**) Distribution of cis-acting elements on *CDPK* upstream, represented by rectangles of different colors. (**B**) *CDPK* gene expression profiles in *D. officinale* in response to CK (untreated leaves), SA (salicylic acid, 10 µM), ABA (abscisic acid, 0.2 µM), auxin (IAA, 2 µM), and JA (jasmonic acid, 10 µM). Data for the transcript levels of *DoCDPK*s with different hormone treatment using the FPKMs (fragments per kilobase of exon model per million mapped fragments) value that downloaded from the Biodiversity Data Center (iflora.cn (accessed on 12 September 2021)). A heatmap was generated using the Genesis software. Parameters were set as log 10 transformation, and genes were normalized. High, moderate, and low expression levels of one gene were shown in red, black, and green, respectively, with different treatments.

Previous studies have reported that several hormone stimuli regulate the expression of *CDPKs*. Therefore, we predicted cis-acting elements in the promoter regions (2000 bp upstream of the start codon) of *CDPK* genes. Although the distribution of cis-acting elements in the promoter regions of *CDPK*s (Figure 4A) varied widely, all *CDPK*s contained elements related to hormone responsiveness. The promoters of more than half of the *CDPK*s had elements involved in response to abscisic acid (ABA), jasmonic acid (JA), and auxin (indole acetic acid, IAA). In addition, 6 out of 24 genes had elements related to salicylic acid (SA) response.

Next, we explored the expression patterns of *CDPK* genes in the leaves of plants under different hormone treatments using data downloaded from the internet. The results of quantitative-polymerase chain reaction (q-PCR) of *DoCDPK6-1*, *DoCDPK11-1* and *DoCDPK29* showed the same trend as the heat map (Figure S2). Although gene transcript levels in leaves were at moderate to low levels under natural conditions, these changed markedly after spraying with different hormones. The transcript levels of all *CDPK* genes were altered at 3 and 6 h following hormone treatment. The majority of genes were upregulated at 3 h, followed by a gradual return to the initial level at 6 h after hormone treatments. Of the 24 *CDPK* genes, 20, 15, 21, and 17 genes were upregulated following treatment with SA, ABA, IAA, and JA, respectively. After treatment with SA, *CDPK*s were upregulated, with 10 *CDPK* genes showing peak expression. Most of them had SA-response elements in their promoter regions. Similarly, most of the upregulated genes in response to JA had cis-acting elements in their promoter regions that were involved in JA response. These results suggested that different genes with different cis-acting elements in their promoter regions participate in distinct pathways to regulate stress resistance in *D. officinale*.

### 2.6. Interaction between Rbohs and CDPKs

**Figure 5:**
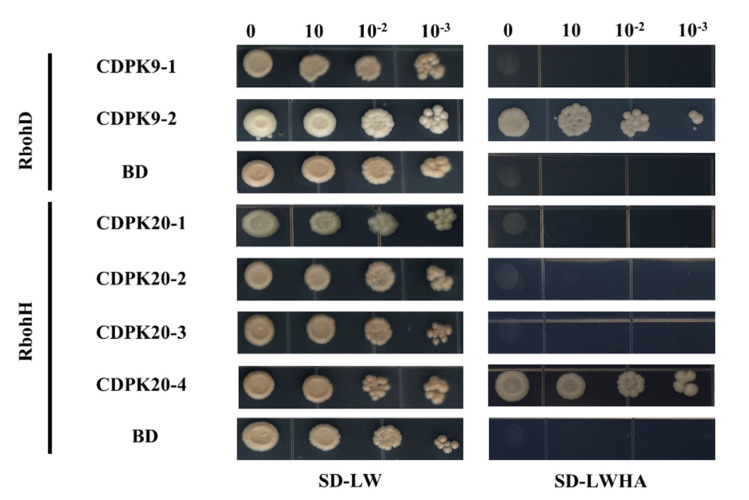
Yeast two-hybrid analysis of the interaction between certain CDPK and Rboh proteins in *D. officinale*. The yeast cells of strain AH109, containing the target plasmid combinations, were grown on a nonselected SD-leucine-tryptophan (SD-LW) or selective SD-leucine-tryptophan-histidine-adenine (SD-LWHA) media. BD is the empty PGBDT7 vector. Here, 0, 10^−1^, 10^−2^, and 10^−3^ represent the dilution multiples of the bacterial solution.

**Figure 6:**
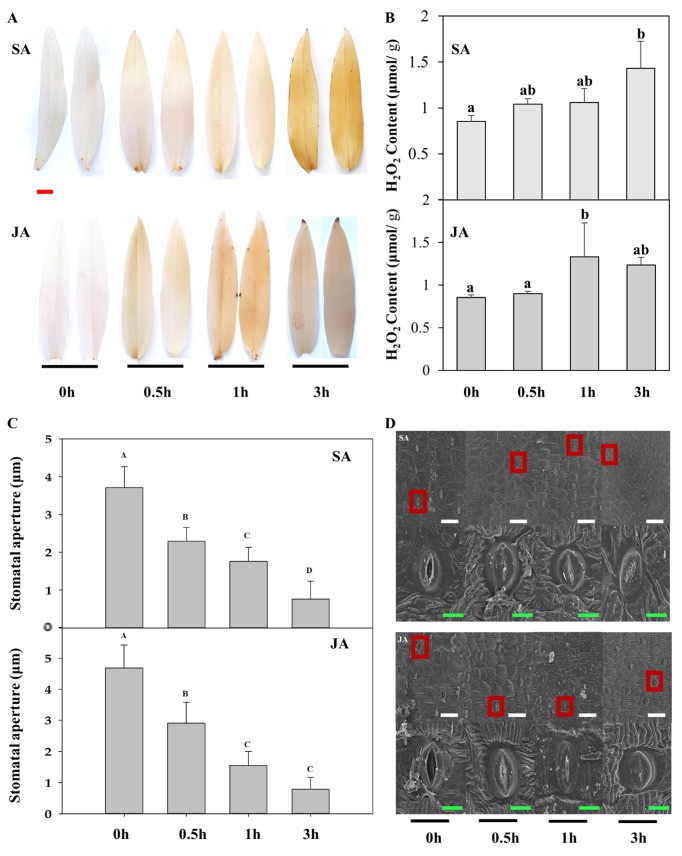
H_2_O_2_ content and changes in stomata aperture in response to treatment with salicylic acid (SA, 10 µM) and JA (jasmonic acid, 10 µM). (**A**) H_2_O_2_ staining with DAB (3,3-diaminobenzidine) in SA/JA treatments. The red bar in the upper image means 0.5 cm. (**B**) H_2_O_2_ content in leaves of plants treated with SA/JA. Means denoted by different letters are significant (*p* < 0.05). (**C**) Effect of SA/JA treatment on the stomatal aperture of *D. officinale*. Five leaves in the same general location were used to record stomatal aperture, and 15 stomata were observed for each sample. Date were analyzed by SigmaPlot. Means denoted by different letters are significant (*p* < 0.01). (**D**) Phenotype of stomatal aperture. Data were collected from three independent experiments. The white bar in the upper images means 100 μm. The figure shown below is an enlargement of that in upper red box. The green bar in the images means 30 μm.

Rboh proteins are known substrates of CDPK and are related to ROS generation under stress. Previous results have shown that the expression of *DoCDPK9-1*/*-2* and *DoCDPK20-1*/*2*/*3*/*4* was increased after treatment with hormones. Yeast two-hybrid analyses (Figure 5) were conducted to assess the interaction between these proteins in *D. officinale*. In addition, the content of H_2_O_2_ (Figure 6A,B) and changes in stomatal aperture (Figure 6C,D), which are related to stress resistance, were determined. To clarify the relationship between stomatal behavior and hormone treatments, H_2_O_2_ in leaves was detected by 3,3-diaminobenzidine (DAB) staining and micro hydrogen peroxide(H_2_O_2_) assay kit (Solarbio, Beijing, China). The yeast two-hybrid results showed that DoCDPK9-1 and DoCDPK20-4 interacted with RbohD and RbohH, respectively. In addition, no H_2_O_2_ content was accumulated in the control, whereas hormone treatments resulted in H_2_O_2_ accumulation in the leaves of plants, with peak levels at 3 and 1 h after the spraying with SA and JA, respectively. Scanning electron microscopy analyses of stomata revealed that the stomatal apertures were larger after treatments with SA and JA, followed by a gradual decrease and finally closure at 3 h after hormone treatments.

## 3. Discussion

The number of *CDPK* genes varies widely among different plant species. Diploid plants such as *A. thaliana*, rice, and soybean have 34, 29, and 50 *CDPK* genes, respectively [5,37,38]. Here, we identified 24 *CDPK* genes and designated them as *DoCDPKs*. Similar to *CDPKs* in other species, *DoCDPKs* form four groups, as confirmed by the phylogenetic study (Figure 1). Groups 1 and 2 of *D. officinale CDPKs* had 9 and 6 members, respectively, compared with 10 and 13 in *A. thaliana*; 11 and 8 in rice; and 17 and 15 in soybean. This was consistent with the observation in other species, the number of *CDPKs* largely depends on the number of genes in groups 1 and 2 [38]. Moreover, studies on *CDPK* genes in rice revealed a greater probability of intron loss than intron gain [39,40]. Similar to the genetic structure of *CDPKs* in populus, grape, and maize, *DoCDPKs* in group 1 had 6–9 introns, and those in group 4 had the highest number of introns [21,41,42]. Therefore, we assumed that *CDPK* genes in group 4 were more ancient than those in other groups. Motif prediction analyses revealed more gene motifs in groups 1–3 than in group 4; the number of motifs among the four groups varied. Thus, we hypothesized that *DoCDPKs* have evolved diverse functions, with genes in the same group having similar functions and those in different groups having different functions. This pattern of functional diversity has also been detected for the *CDPKs* of pepper, rice, and maize [20,43,44]. Altogether, these results suggest that gene function exerts a feedback effect on the copy number of genes in a family [45].

Gene duplication has been observed in flies as early as 1963 [46]. Subsequent studies reported that it played a crucial role in the adaptation of species to the environment [47]. Species evolution can be detected using the Ks/Ka ratio [48]. We calculated this index for homologous gene pairs to explore the selection of *DoCDPKs*. Similar to the *CDPKs* in turnip and cucumber, those in *D. officinale* displayed a low Ka/Ks ratio (less than 1, Table 2), indicating that *DoCDPKs* had undergone negative selection [49,50]. The Ks values differed among gene pairs, indicating the evolution rate varied in homologous genes which could result in functional diversification. *CDPKs* are known to regulate diverse biological processes, including responses to hormones [51,52,53,54,55]. In several plant species, *CDPK* transcript levels changed with hormone treatments, including JA and SA [56,57,58]. The majority of *DoCDPKs* in our study were upregulated following the SA/JA treatment (Figure 4B), whereas homologous genes showed varied expressions, indicating functional divergence among *CDPK*s. This finding is consistent with the *CDPK* expression profiles in grapevine and *DcSDG* gene in *Dendrobium catenatum*, suggesting functional divergence in paralogs [19,59]. Further study of cis-acting elements (Figure 4A) revealed differences between homologous genes in terms of the presence of stress-responsive elements and elements involved in the regulation of growth and development in their promoter regions. These findings indicated a functional divergence of *DoCDPKs*.

The interaction between *CDPK* and *Rboh* regulates the rapid generation of ROS in response to stress stimuli in plants [60,61]. In potatoes, *StCDPK5* interacts with *StRbohC* to increase pathogen resistance via ROS burst [29]. Here, we found that DoCDPK 9-2 and DoCDPK 20-4 interacted with Rboh D and Rboh H separately (Figure 5); however, their homologous gene DoCDPK9-1 and DoCDPK20-1/-2/-3 did not interact with Rbohs. These results, in combination with the upregulated expression of homologous gene pairs *CDPK9-1/-2* and *CDPK20-1/-2/-3/-4* following JA/SA treatments, suggested a different role of homologous gene pairs in SA/JA-induced ROS accumulation, indicating that the function of *CDPK9-1/-2* and *CDPK 20-1/-2/-3/-4* was differentiated. AtCDPK1 interacted with AtRbohD to regulate *Rboh*-mediated ROS generation and protect *A. thaliana* from DC3000 [62]. Here, we found that H_2_O_2_ content increased in hormone-treated leaves (Figure 6A,B), and the stomata closed gradually (Figure 6C,D). The timely closure of the stomata protects plants from pathogens and pests [63]. Our further study found the stomal aperture decreased following hormone treatments (Figure 6C,D). These findings suggest that interactions of DoCDPKs and DoRbohs in JA/SA induced stomatal closure via the H_2_O_2_ pathway. This finding is consistent with the result that the overexpression of *VpCDPK9* enhanced the grapevine resistance to powdery mildew due to the increased production of SA and excess accumulation of H_2_O_2_ in the epidermal cells. Similarly, the expression of stress-responsive genes, including *AtRboh*, was increased in the *CsCDPK20* transgentic *A. thaliana* [64,65].

## 4. Methods

### 4.1. Identification of Members of CDPK Gene Family in D. officinale

Genomic sequences were obtained from the website (Index of/genomes/all/GCF/001/605/985/GCF_001605985.2_ASM160598v2 (nih.gov) (accessed on 5 August 2021)). Proteins containing PF00069 (protein kinase domain) and PF13499 (EF-hand calcium-binding domains) [42] were identified from the *D. officinale* genome using a Simple HMM Search in TBtools. The number of EF-hand domains was predicted by SMART (embl-heidelberg.de (accessed on 15 August 2021)) [66], the molecular weight, theoretical isoelectric point, grand average of hydropathicity, and myristoylation sites were predicted using ExPASy (https://web.expasy.org (accessed on 21 August 2021)), and palmitoylation and N-terminal acetylation were detected using CCS-Palm 4.0 GSP-PAIL 2.0 (biocuckoo.org (accessed on 21 August 2021)) [67,68].

### 4.2. Phylogenetic and Gene Structure Analyses

The *A. thaliana CDPK* genes sequences were downloaded from The Arabidopsis Information Resource (TAIR, arabidopsis.org (accessed on 5 August 2021)). Both *D. officinale* and *A. thaliana* CDPK protein sequences were aligned using MAFFT 7.0. Next, MEGAX version 7.0 was used to construct a phylogenetic tree using the neighbor-joining (NJ) method [69]. Gene exon/intron structures were analyzed using tools available at the Gene Structure Display Server (gao-lab.org (accessed on 21 August 2021)) [70].

### 4.3. Evolutionary and Conserved Motif Analyses

Gene pairs with close genetic relationships were identified, and the synonymous (Ks) and nonsynonymous (Ka) substitution rates were calculated using TBtools. The amino acid sequence identity between each pair of DoCDPK proteins was calculated using the DNAMAN software. The conserved motifs of DoCDPKs were predicted using tools available at MEME (meme-suite.org (accessed on 14 November 2021)).

### 4.4. Cis-Acting Element Prediction and Analyses of Gene Expression

Data for the transcript levels of *DoCDPK* genes in ten tissues including the flower, sepal, labellum, pollinium, gynostemium, stem, leaf, root, green root tip (root tip), and white part of the root (part root), and treatments with different hormones (foliar sprayed with 10 µM SA, 0.2 µM ABA, 2 µM IAA, and 10 µM JA) were downloaded from OrchidBase (ncku.edu.tw (accessed on 12 September 2021)) and Biodiversity Data Center (iflora.cn (accessed on 15 September 2021)), respectively [71]. A heatmap was constructed using the Genesis software [72]. Sequences 2000 base pair upstream of the coding sequences of *DoCDPK*s were extracted using TBtools and submitted to PlantCare (ugent.be (accessed on 17 September)) to predict cis-acting elements.

### 4.5. RNA Extraction and Analysis of Quantitative Real-Time PCR

The total RNA of untreated 6 month-old catenatum leaves (CK) and leaves sprayed with 10 µM SA, 0.2 µM ABA, 2 µM IAA, and 10 µM JA at 3 and 6 h were isolated using method described by Ksenija Gassic [73]. The RNA quality was determined using NanoDrop 1000 spectrophotometer (NanoDrop Technologies, Wilmington, DE, USA). Afterward, 0.5 μg of RNA was used for first-strand complementary DNA (cDNA) synthesis by Superscript III reverse transcriptase (Invitrogen, Waltham, MA, USA), following the manufacturer’s instructions. Primers for q-PCR (Table S2) were designed using Primer-BLAST (Primer designing tool (nih.gov) (accessed on 14 November 2021)), offering the following parameters: 100–200 base pair of PCR product size, Refseq mRNA database, 57–63 °C primer melting temperatures, and *Dendrobium catenatum* (TaxID: 906689) for “Organism”. Each sample included three biological and technical replicates. PCR reactions were performed under the following conditions: 40 cycles of 5 s at 95 °C, 15 s at 60 °C, and 34 s at 72 °C using FastStart Universal SYBR Green Master (Rox, Roche, Indianapolis, IN, USA).

### 4.6. Yeast Two-Hybrid Analyses

The full-length coding sequences of *DoCDPK9-1/9-2/20-1/20-2/20-3/20-4* were subcloned into the PGBKT7(BD) vector (Generay Biotech Co. Ltd., Shanghai, China). Three Rbohs, named DoRboh D/F/H, were obtained from *D. officinale* with the *A. thaliana* Rboh as the query and subcloned into the PGADT7(AD) vector (Generay Biotech Co. Ltd., Shanghai, China). The primers are shown in Table S3. The transformed plasmids harboring *CDPK* and *Rboh* sequences were introduced into the yeast strains Y2H and Y187, respectively. After being plated on two kinds of media, that nonselective media (SD-leucine-tryptophan) and selective media (SD-leucine-tryptophan-histidine and SD-leucine-tryptophan-histidine-adenine), were performed according to the method described by Wang [45].

### 4.7. Determination of Hydrogen Peroxide Content and Stomatal Aperture in Leaves

*Dendrobium officinale* was cultivated in a 5:1 (*w*/*w*) soil: sand mixture in a greenhouse at 24 °C with 60–80% relative humidity and 16 h of photoperiod (daytime, 06:00–20:00). Six month-old catenatum leaves were sprayed with 10 µM SA or JA separately and sampled at 0, 0.5, 1, and 3 h after the treatment; 0 h was set as the control, according to the method described by Zhang [74]. Leaf samples were soaked in 3,3-diaminobenzidine (DAB, 1 mg/mL, PH 3.8) for 8 h, and pigments were removed using boiling alcohol before observing the DAB staining pattern. The H_2_O_2_ content was measured following the manufacturer’s instructions using the H_2_O_2_ assay kit (Solarbio, China). Other leaf samples were observed from stomata, and the stomatal aperture was measured under a scanning electron microscope using the method described by Yang [75]. Fresh leaves were fixed with 1% *v/v* glutaraldehyde for at least 24 h at 4 °C and subsequently substituted with a series gradient concentration of alcohol (50%, 70%, 85%, 95%, and 100%), each for 1 h before being subjected to critical-point-drying using liquid carbon dioxide and coated with gold and palladium. Five leaves in the same general location were used to observe the stoma per treatment under a scanning electron microscope. Fifteen stomata were measured for each sample.

## 5. Conclusions

As one of the crucial signal molecules in the plant stress response, members of *CDPK* family have been studied in several species. Although no study has been reported in *D. officinale*, we identified members of the *CDPK* gene family in *D. officinale* and explored their functions in response to SA and JA stress. We identified 24 members in the *D. officinale CDPK* gene family. The differences in their evolution rates and expression profiles were indicative of functional divergence. Among them, *DoCDPK 9-2*, and *DoCDPK 20-4* were upregulated by SA or JA treatment. In addition, they interacted with *Rboh D/H* to decrease the stomatal aperture by regulating the accumulation of H_2_O_2_ in the leaves in response to stress.

## Figures and Tables

**Table 1:** Characteristics of CDPK in *Dendrobium officinale*.

Gene Name	ACC.NO.	CDS (bp)	EF Hands	MW (Kda)	PI	GRAVY	M	P	T
*DoCDPK6-1*	XP_020676095.1	1665	4	61.72	5.74	−0.228	N	Y	N
*DoCDPK6-3*	XP_028549092.1	1665	4	62.11	5.55	0.288	N	Y	N
*DoCDPK6-2*	XP_020702402.1	1683	4	61.84	5.45	−0.254	Y	Y	N
*DoCDPK11-1*	XP_020685368.1	1494	4	56.03	5.43	−0.339	N	Y	N
*DoCDPK11-2*	XP_020689725.1	1488	4	55.64	5.39	−0.315	N	Y	N
*DoCDPK20-1*	XP_020694473.1	1752	4	64.82	5.15	−0.344	N	Y	N
*DoCDPK20-4*	XP_020692403.1	1725	4	63.92	5.51	−0.313	N	Y	N
*DoCDPK20-3*	XP_020684094.1	1773	4	65.7	5.15	−0.318	N	Y	N
*DoCDPK20-2*	XP_028550805.1	1764	4	65.73	5.15	−0.318	N	Y	N
*DoCDPK34*	XP_020691369.2	1440	4	54.19	5.71	−0.385	N	Y	N
*DoCDPK3-2*	XP_020673496.1	1536	4	57.19	5.71	−0.385	N	Y	N
*DoCDPK3-1*	XP_020700773.1	1566	4	58.17	5.84	−0.411	N	Y	N
*DoCDPK29*	XP_020691445.1	1563	4	58.58	6.23	−0.365	Y	Y	N
*DoCDPK9-2*	XP_020688324.1	1578	4	58.33	5.94	−0.432	Y	Y	N
*DoCDPK9-1*	XP_020698035.1	1605	4	59.67	6.03	−0.477	Y	Y	N
*DoCDPK24*	XP_020688637.2	1302	4	49.42	5.35	−0.398	N	Y	N
*DoCDPK13-1*	XP_020684921.1	1329	3	60.46	6.14	−0.457	N	Y	N
*DoCDPK13-2*	XP_028548644.1	930	3	35.07	5.02	−0.468	N	Y	N
*DoCDPK7*	XP_020678675.1	1608	3	60.13	6.38	−0.414	N	Y	N
*DoCDPK8-2*	XP_020682170.1	1611	4	60.3	6.46	−0.476	Y	Y	N
*DoCDPK8-1*	XP_020698328.1	1608	3	60.35	6.32	−0.464	Y	Y	N
*DoCDPK28-1*	XP_020702698.1	1605	3	60.63	6.7	−0.419	Y	Y	N
*DoCDPK16*	XP_028552413.1	1716	3	64.42	8.08	−0.585	Y	Y	N
*DoCDPK28-2*	XP_020676625.1	1545	4	57.52	5.84	−0.204	N	Y	N

ACC.NO., Genebank Accession Number. CDS, length of CDS. EF hands, number of EF hands. GRAVY, grand average of hydropathicity; M, myristoylation site, P, palmitoylation site; T, N-terminal acylation; N, NO; Y, YES.

**Table 2:** Estimated natural selection of *D. officinale CDPK* homologous gene pairs.

Seq_1	Seq_2	Identity (%)	Ka	Ks	ω
*DoCDPK6-1*	*DoCDPK6-3*	86.10	0.0942	1.0008	0.0941
*DoCDPK6-1*	*DoCDPK6-2*	81.13	0.0751	0.6947	0.1081
*DoCDPK6-2*	*DoCDPK6-3*	79.86	0.0895	0.8868	0.1009
*DoCDPK11-1*	*DoCDPK11-2*	87.53	0.0630	0.9029	0.0698
*DoCDPK20-3*	*DoCDPK20-4*	81.19	0.0909	0.7991	0.1138
*DoCDPK20-3*	*DoCDPK20-2*	80.34	0.1071	1.0135	0.1057
*DoCDPK20-2*	*DoCDPK20-4*	78.23	0.1137	0.8833	0.1287
*DoCDPK3-2*	*DoCDPK3-1*	69.60	0.1943	1.5219	0.1277
*DoCDPK9-2*	*DoCDPK9-1*	83.02	0.0738	0.7152	0.1033
*DoCDPK13-1*	*DoCDPK13-2*	49.72	0.1970	1.1168	0.1708
*DoCDPK7*	*DoCDPK8-2*	86.19	0.0793	0.8846	0.0897
*DoCDPK7*	*DoCDPK8-1*	85.23	0.0848	0.8835	0.0960
*DoCDPK8-2*	*DoCDPK8-1*	87.50	0.0659	0.8111	0.0813
*DoCDPK28-1*	*DoCDPK16*	70.23	0.1502	2.1678	0.0693

Ka, nonsynonymous substitution rate; Ks, synonymous substitution rate; ω, Ka/Ks.

## Data Availability

Not applicable.

## Supplementary Materials

The following supporting information can be downloaded at: https://www.mdpi.com/article/10.3390/ijms23031298/s1.
