# Supplementary material for: Genome-Wide Survey Indicates Diverse Physiological Roles of Dendrobium officinale Calcium-Dependent Protein Kinase Genes
Source: Int J Mol Sci. 2022 Jan 24;23(3):1298. doi: 10.3390/ijms23031298 (PMC8835911; doi:10.3390/ijms23031298)
Supplement: Supplementary file 1 [file ijms-23-01298-s001.zip › ijms-1487512-supplementary.pdf]

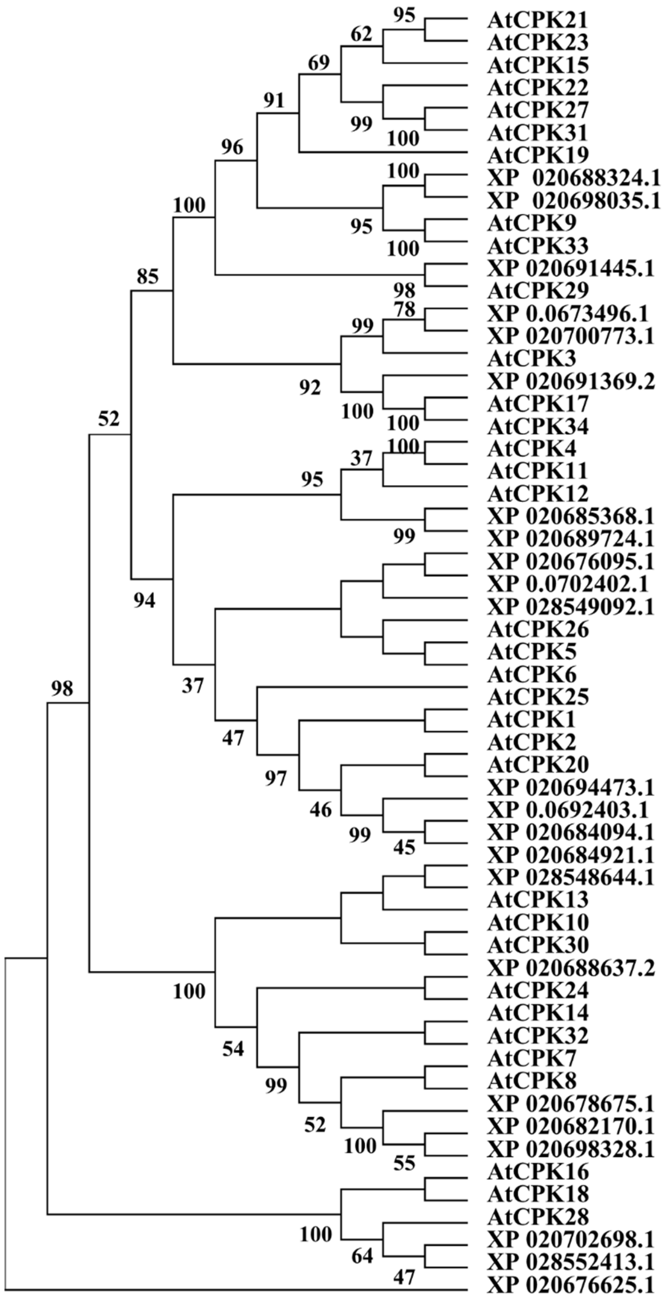

Figure S1. Phylogenetic relationship of CDPKs in *Dendrobium officinale* and *Arabidopsis thaliana*. A neighbor-joining tree was created using full-length protein sequences of *CDPKs* using MEGAX 7.0 with 1000 bootstrap replicates. Numbers beside branches indicate bootstrap support.

Table S1. Identity of unnamed CDPK in *D.officinale* and *A.thaliana*

**Gene name in *D.officinale***	**Gene name in *A.thaliana***	**Identity(%)**	**Gene name in article**
XP_020688324.1	AtCPK9	72.83	DoCDPK9-2
XP_020698035.1	AtCPK9	72.95	DoCDPK9-1
XP_020688324.1	AtCPK33	70.79	
XP_020698035.1	AtCPK33	72.71	
XP_020691445.1	AtCPK3	72.02	DoCDPK3-1
XP_020700773.1	AtCPK3	63.47	DoCDPK3-2
XP_020691369.2	AtCPK34	73.47	DoCDPK34
XP_020691369.2	AtCPK17	72.97	
XP_020685368.1	AtCPK12	72.85	
XP_020685368.1	AtCPK11	74	DoCDPK11-1
XP_020685368.1	AtCPK4	72.78	
XP_020689724.1	AtCPK12	72.69	
XP_020689724.1	AtCPK11	72.95	DoCDPK11-2
XP_020689724.1	AtCPK4	71.54	
XP_020676095.1	AtCPK26	74.42	
XP_020676095.1	AtCPK5	77.36	
XP_020676095.1	AtCPK6	79.17	DoCDPK6-1
XP_020702402.1	AtCPK26	73.29	
XP_020702402.1	AtCPK5	74.08	
XP_020702402.1	AtCPK6	76.03	DoCDPK6-3
XP_028549092.1	AtCPK26	72.57	
XP_028549092.1	AtCPK5	75.40	
XP_028549092.1	AtCPK6	77.01	DoCDPK6-2
XP_020694473.1	AtCPK20	77.01	DoCDPK20-1
XP_020684094.1	AtCPK20	68.17	DoCDPK20-3
XP_028550805.1	AtCPK20	69.05	DoCDPK20-2
XP_020692403.1	AtCPK20	68.01	DoCDPK20-4
XP_020684921.1	AtCPK13	79.67	DoCDPK13-1
XP_028548644.1	AtCPK13	48.11	DoCDPK13-2
XP_020678675.1	AtCPK7	75.46	DoCDPK7
XP_020678675.1	AtCPK8	75.19	
XP_020682170.1	AtCPK7	76.02	
XP_020682170.1	AtCPK8	76.72	DoCDPK8-2
XP_020698328.1	AtCPK7	77.84	DoCDPK8-1
XP_020698328.1	AtCPK8	77.84	
XP_020702698.1	AtCPK16	70.40	
XP_020702698.1	AtCPK18	66.43	
XP_020702698.1	AtCPK28	74.53	DoCDPK28-1
XP_020692403.1	AtCPK16	25.29	
XP_020692403.1	AtCPK18	24.48	

XP_020692403.1	AtCPK28	26.50	DoCDPK28-2
XP_028552413.1	AtCPK16	73.78	DoCDPK16
XP_028552413.1	AtCPK18	66.56	
XP_028552413.1	AtCPK28	72.50	

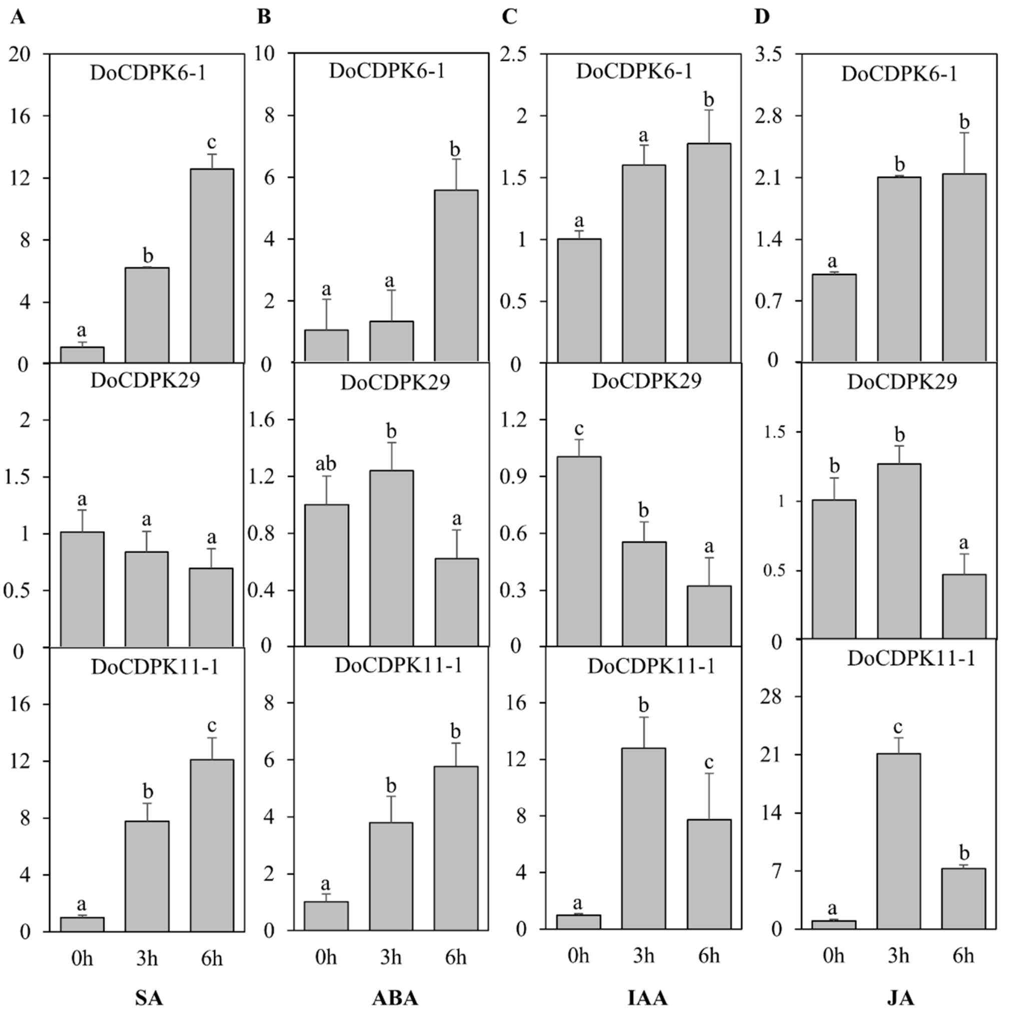

Figure S2. Results of quantitative real-time PCR (q-PCR) of DoCDPK6-1, 11-1 and 29 with SA, ABA, IAA and JA treatment at 0, 3 and 6 hours. (A) Results of quantitative real-time PCR (q-PCR) of DoCDPK6-1, 11-1 and 29 with SA treatment at 0, 3 and 6 hours. (B) Results of quantitative real-time PCR (q-PCR) of DoCDPK6-1, 11-1 and 29 with ABA treatment at 0, 3 and 6 hours. (C) Results of quantitative real-time PCR (q-PCR) of DoCDPK6-1, 11-1 and 29 with IAA treatment at 0, 3 and 6 hours. (D) Results of quantitative real-time PCR (q-PCR) of DoCDPK6-1, 11-1 and 29 with JA treatment at 0, 3 and 6 hours. Means denoted by different letters are significantly (P<0.05).

Table S2. Primers used for q-PCR

Gene name	Forward primer (5′-3′)	Reverse primer (5′-3′)
DoCDPK6-1	CCCCACTACAATCCGTGTCC	CCCCACTACAATCCGTGTCC
DoCDPK12	CAATCGTTGCGGTCGTTGAG	AGGGGCTTCCCACTACATCA
DoCDPK29	TTCAGCATCTTACGGGGCAA	GCTCCGAATAGCTCCCCTTG
Actin	GAGACCTTCAATGCCCCTGCTAT	GGCTGACACCATCACCAGAATCC

Table S3. Primers used for yeast two-hybrid assay.

Gene name	Forward primer (5′-3′)	Reverse primer (5′-3′)
DoCDPK9-1	GAGGACCTGCATATGATGGGAGCTTGCTGCGGCAAA	CAGGTCGACGGATCCAATAATCTTAGCTGGTTGCTGAAA
DoCDPK9-2	GAGGACCTGCATATGATGGGATCTTGCTGCGGAAAA	CAGGTCGACGGATCCGGCATGCCTAGCTGGTTGCGGAAG
DoCDPK20-1	GAGGACCTGCATATGATGGGTAACAACTGCGTCGGT	CAGGTCGACGGATCCTTTTGCTGGCTCTTTCTGCCC
DoCDPK20-2	GAGGACCTGCATATGATGGGGAACACTTGTGTCGG	CAGGTCGACGGATCCGCCAACCTTCAAAGCTTCTCT
DoCDPK20-3	GAGGACCTGCATATGATGGGGAATACATGTGTTGGTCC	CAGGTCGACGGATCCAAGCATCAGGGCCTCCCTAAG
DoCDPK20-4	GAGGACCTGCATATGATGGGAAATACATGTGTCGGCCC	CAGGTCGACGGATCCAACAAGCCTCAAAGCCTCCCT
DoRboh D	GATTACGCTCATATGATGAAGAGATTGGGCGGCTCC	GAGCTCGATGGATCCGAAGTTCTCCTTGTGGAAATC
DoRboh H	GATTACGCTCATATGATGGGATCAACGGCCTACGTC	GAGCTCGATGGATCCGAAGTTCTCCTTGTGAAAAT
DoRboh F	GATTACGCTCATATGATGAAGGGATTCGCGACGCAC	GAGCTCGATGGATCCGAAATGCTCCTTGTGGAACTC
